# Cardiac complications in community-acquired pneumonia and COVID-19

**DOI:** 10.7196/AJTCCM.2020.v26i2.077

**Published:** 2020-05-14

**Authors:** C Feldman

**Affiliations:** Department of Internal Medicine, Faculty of Health Sciences, University of the Witwatersrand, Johannesburg, South Africa

**Keywords:** Cardiac, Cardiovascular, Community-acquired pneumonia, COVID-19, pneumococcus, Pneumonia

## Abstract

Community-acquired pneumonia (CAP) remains a global health problem with significant morbidity and mortality. Much recent published literature about the infection has indicated that a substantial number of patients with CAP, particularly those ill enough to be admitted to hospital, will suffer a cardiovascular event. While these may include events such as deep venous thrombosis and stroke, most of the events involve the heart and include the occurrence of an arrhythmia (most commonly atrial fibrillation), new onset or worsening of heart failure and acute myocardial infarction. While such cardiac events may occur, for example, in all-cause CAP and CAP due to influenza virus infection, and more recently described with the SARS-CoV-2 pandemic, a significant amount of research work has been investigating the pathogenic mechanisms of these cardiac events in patients with CAP due to *Streptococcus pneumoniae* (pneumococcus) and, more recently, COVID-19 infections. Such research has identified a number of mechanisms by which these microorganisms may cause cardiovascular events. Importantly, these cardiac events appear not only to be associated with in-hospital mortality, but they also appear to contribute to longer-term mortality of patients with CAP, even after their discharge from hospital. This review will focus initially on studies of cardiovascular events in all-cause CAP and pneumococcal CAP, excluding COVID-19 infection, and then address similar issues in the latter infection.

## Background

Community-acquired pneumonia (CAP) continues to be associated with considerable morbidity and mortality in patients throughout the world. A number of recent reviews on the topic of CAP attest to the fact that, despite intensive research activity in this field over many years and all the advances that have been made in medical management during this time, such as the development of potent antimicrobial chemotherapy, improvements in medical and nursing care, and even the establishment of intensive care unit facilities, this infection remains associated with significant complications, healthcare costs and mortality.^[1–4]^ All the reviews make mention of the fact that in patients with CAP there is not only a significant short-term mortality (such as in-hospital or thirty-day mortality) but that these patients have impaired long-term outcomes, even after apparent successful treatment of the acute infection, and that these impaired outcomes may span several years following hospital discharge.^[1–4]^ Furthermore, long-term mortality in patients who have been discharged following hospital admission for CAP is higher than in patients admitted to hospital for other medical conditions, even after adjustments are made for confounding variables. A number of factors have been described as apparent predictors of long-term mortality, which may occur even in the absence of underlying comorbid conditions, and prominent among these predictors is the occurrence of cardiovascular complications.^[1–4]^

## Cardiovascular complications in allcause CAP and pneumococcal CAP

A number of reviews,^[3–10]^ systematic reviews and meta-analyses^[11]^ and various studies ^[12,16]^ (by no means a comprehensive list of all publications) have described the occurrence of cardiovascular complications in all-cause and pneumococcal CAP. Among the early studies was one by Spodick *et al*.,^[17]^ who studied 150 patients hospitalised with acute myocardial infarction (AMI) and 150 appropriately matched controls, and noted that there was an increased risk (odds ratio (OR) 2.2; p<0.02) of minor acute respiratory symptoms in patients with AMI. The authors suggested that there was a need to investigate whether there was a relationship between these infections, assumed to be of viral origin, and AMI. Interestingly, and following the consideration that most studies noting that respiratory infections were associated with an increased short-term risk of AMI were based on raised troponin and/or electrocardiographic (ECG) changes, a recent study confirmed angiographically that this association was indeed present.^[18]^ Another early study was that of Seedat *et al*. ^[12]^ who prospectively studied consecutive patients with CAP hospitalised at Chris Hani Baragwanath Academic Hospital, who had no prior coexisting cardiorespiratory illness. These investigators performed, among a number of other investigations, electrocardiograms (ECGs), echocardiography and measurement of serum enzyme levels (creatinine kinase (CK), including CK-MB fraction and lactate dehydrogenase (LDH)). The authors noted that a number of ECG changes occurred in these patients, which returned to normal after a mean of two days in those who survived. The so called S1, Q3, T3 pattern on ECG was associated with CK-MB leak, hypoxia and a high Simplified Acute Physiology Score (SAPS). The presence of P pulmonale, right axis deviation and clockwise rotation correlated with hypoxia and a high SAPS score. Clockwise rotation also correlated with raised serum LDH and CK-MB levels and high pulmonary artery pressures. The mortality was 10.8% and, while there was no association between ECG changes and mortality, raised LDH, CK and CK-MB levels correlated with need for intensive care unit admission and mortality. An early study by Musher *et al*. ^[13]^ was the first to document cardiac changes specifically in CAP due to *Streptococcus pneumoniae* (pneumococcus).

The findings from the various publications on cardiac complications in CAP^[5,9-11,13-15,19-21]^ are described here in more detail. While deep venous thrombosis and stroke may occur with CAP, most cardiovascular complications involve the heart and include the occurrence of arrhythmia (most commonly atrial fibrillation), new or worsening heart failure and AMI. These complications may occur in up to 30% of hospitalised patients with all-cause CAP. Musher *et al*. ^[13]^ documented cardiac events in almost 20% of cases with pneumococcal CAP. These complications are more common in older individuals, those with more severe pneumonia and in those with pre-existing cardiovascular events; however, it is important to note that they can occur in cases with no underlying risk factors. Most of these events occur within the first week of illness although some 50% are noted within the first 24 hours. Importantly, while these events are associated with short-term mortality, they may also be associated with long-term mortality, with episodes of cardiovascular events occurring with increased frequency after hospital discharge, extending over a 10-year period, although most appear to occur within the first year.

## Possible pathogenic mechanisms of cardiac events in pneumococcal CAP

Significant progress has been made in understanding the mechanisms of cardiac complications in patients with CAP, with much of these investigations focusing on the pathogenesis of these complications in relation to pneumococcal infections. A number of possible mechanisms have been described, some of which are described below.

### Pneumococcal microlesions in the heart

Studies in experimental animal models with bacteraemic pneumococcal infection have documented the occurrence of microscopic lesions (microlesions) filled with pneumococci in the heart muscles, but without the presence of infiltrating immune/inflammatory cells.^[22,23]^ Similar microlesions have been noted in humans who succumbed to invasive pneumococcal disease, but these were devoid of bacteria, possibly as a consequence of antibiotic therapy during the acute illness. Factors required for the formation of these microlesions included the presence of the pneumococcal pore-forming toxin, pneumolysin, and for bacterial translocation into the heart, this required the pneumococcal adhesion receptor, choline binding protein A (CbpA), as well as the host ligand’s laminin receptor and platelet-activating receptor (PAFR). Importantly, these lesions were noted in the experimental animal model to mature in the presence of antibiotic treatment, with infiltration of immune cells and deposition of collagen with scar formation. It was suggested by the authors that the occurrence of this scarring in the heart might be responsible not only for short-term effects but also for long-term cardiac events following pneumococcal infection. Similarly, in a non-human primate model, the pneumococcus was documented to invade the myocardium, causing cardiac myocyte death via necroptotic and apoptotic mechanisms, and cardiac dysfunction, which was followed by scarring with antibiotic treatment.^[24]^

### Pneumolysin

In addition to the role of pneumolysin described above, it has also been documented that pneumolysin (and possibly other pneumococcal toxins, such as hydrogen peroxide) may directly cause cardiac myocyte death.^[25,26]^ Furthermore, pneumolysin has been shown to kill infiltrating macrophages by necroptosis, which appears to be strain specific and may be the reason why the microlesions contain few immune and inflammatory cells.^[26]^ In addition to these direct actions of pneumolysin, this toxin also has an indirect pro-inflammatory/prothrombotic potential, which may be involved in the pathogenesis of cardiac damage and dysfunction (see below). The multifaceted role of pneumolysin in the pathogenesis of myocardial injury in CAP has been reviewed elsewhere.^[27]^

### Platelet activation

Cangemi *et al*. ^[28]^ documented that platelet activation may occur in patients with CAP and that its occurrence appears to be related to the occurrence of myocardial infarction. In their study, the taking of aspirin at a dose of 100 mg/day was not associated with a lower rate of myocardial infarction compared with patients who were not on aspirin. Others have demonstrated, in an experimental animal model, that invasive pneumococcal disease was associated with activation and hyperreactivity of platelets.^[29]^ We have documented that pneumolysin alone activates platelets *in vitro*, causing homotypic aggregation, activates the platelet activating receptor, and also mediates heterotypic aggregation of neutrophils and platelets *in vitro*, thus providing potentially additional mechanisms by which pneumolysin may mediate cardiac events, via platelet and neutrophil activation.^[30,31]^ The mechanisms of platelet activation by the pneumococcus and the role of platelets in CAP have been reviewed elsewhere.^[32]^

## Potential adjunctive therapy for allcause and pneumococcal CAP

Since the mortality of CAP remains exceedingly high, despite early and adequate antimicrobial chemotherapy, it has been suggested that adjunctive therapy may be of importance in cases with severe infection and a number of different adjunctive treatments have been tested in the setting of CAP.^[33]^ Several of these agents may also play a role in attenuating the cardiovascular complications associated with CAP, by targeting aspects associated with the pathogenesis of these cardiac events, a detailed description of which is beyond the current manuscript, but has been extensively reviewed elsewhere.^[6,27]^

One example is the use of antiplatelet agents and there are a number of these available although, at the current time, limited studies have been undertaken in this area.^[6]^ The initial study by Cangemi *et al*.,^[28]^ described above, did not show apparent benefit in reducing the rate of AMI by administration of 100 mg of aspirin per day. The same investigators did, however, show that chronic aspirin use in the elderly was associated with a lower rate of 30-day mortality in hospitalised patients with CAP and a decreased rate of non-fatal cardiovascular events compared with those who were not on aspirin.^[34]^ The authors also showed that in patients with septic shock secondary to CAP, a combination of low-dose aspirin (100 mg/day) and macrolide antibiotics appeared to be associated with improved survival.^[35]^ The authors attributed this to the anti-inflammatory therapy exhibited by both these drugs, as well as the reduction in acute cardiovascular events associated with aspirin therapy. A multicentre, prospective, randomised study documented a reduction in the occurrence of acute coronary syndrome and cardiovascular mortality in patients with CAP treated with aspirin (300 mg aspirin daily for one month) compared with the control group.^[36]^ With regard to current recommendations for anti-platelet therapy, there is still the need for further controlled, definitive intervention trials in this setting.^[6]^

Furthermore, in severely ill patients with CAP, antibiotic therapy combining macrolides with a β-lactam has frequently been shown to be associated with a better outcome than β-lactam therapy alone or even fluoroquinolone monotherapy.^[27]^ The reason for the benefit in adding macrolides is unclear, but macrolides have been documented to have anti-inflammatory/immunomodulatory activity.^[27]^ In addition, as opposed to β-lactam antibiotics, which lyse bacteria and therefore potentiate the release of pneumolysin, macrolides (via their effects on protein synthesis) have been shown by us to have inhibitory effects on pneumolysin production by the pneumococcus, even at sub-inhibitory concentrations and in the setting of macrolide resistance.^[6,27]^ These effects may underpin the benefits of macrolides as adjuncts to β-lactam antibiotics. 

Corticosteroid use has been shown to significantly reduce the mortality in adult patients with severe CAP.^[37]^ People with severe and non-severe CAP who were treated with corticosteroids had lower clinical failure rates (death from any cause, radiographic progression, clinical instability at days 5 - 8).^[37]^ Corticosteroids also reduced time to clinical cure, length of hospital and ICU stay, development of respiratory failure or shock and rates of pneumonia complications. Furthermore, a recent retrospective study documented that in-hospital corticosteroid treatment was associated with a lower incidence of myocardial infarction (MI) in hospitalised adults with CAP.^[38]^ In addition, Cangemi *et al*. ^[39]^ documented in a cohort study, that patients with CAP overproduce 11-dehydro-thromboxane (Tx) B2, which is a reliable marker of platelet activation, and that 11-dehydro-TxB2 levels were independent predictors of AMI (p=0.005). Furthermore, corticosteroids reduced platelet release of TxB2 *in vitro* and urinary excretion of 11-dehydro-TxB2 *in vivo* in patients with CAP. Thus, corticosteroids may play a novel role in decreasing platelet activation in CAP.

While statin use, mainly chronic pre-hospital administration, has been shown in a number of studies to decrease the risk of CAP and/or the mortality in patients who develop CAP – often attributed to their pleiotropic activities – statins have also been shown to attenuate the harmful actions of pneumolysin.^[6]^ A number of additional agents have been investigated, and documented to have activity against pneumolysin, which need to be more fully elucidated in additional studies; these agents are described in detail elsewhere.^[27]^

## Relationship between SARS-CoV-2 infection and cardiovascular events

While much of this review has concentrated on all-cause CAP (in many studies of undetermined microbial aetiology) or specifically pneumococcal CAP, it is important to remember that viral pneumonia, particularly influenza pneumonia, has been documented to be an important cause of AMI.^[40]^ In this regard, it is important to be cognisant of the potential relationships that exist between the pandemic that faces the word at the current time, i.e. SARS-CoV-2 infection (COVID-19) and cardiovascular disease.^[41]^ It is also possible that certain of the issues described above are somewhat different in the case of SARS-CoV-2 infection; however, we are still in a steep learning curve with regard to cardiovascular events in patients during the pandemic.

What we do know is that underlying cardiovascular disease, in addition to hypertension and other comorbid conditions, has been associated with possible increased risk of COVID-19 infection, as well as severe disease and increased mortality,^[41]^ much like what was reported with SARS^[42]^ and MERS-CoV^[43]^ previously. While the early studies of SARS-CoV-2 confirmed the importance of underlying comorbid illness, including cardiovascular disease, there was not much mentioned about the occurrence of acute cardiovascular events during the course of the illness.^[44]^ Later studies noted both that patients with underlying ‘cardiovascular metabolic disease’, were associated with more severe disease and mortality, but also that some 8.0% of cases with COVID-19 infection suffered acute cardiac injury.^[45]^ The incidence of acute cardiac injury was about 13 times higher in critically ill/ICU cases, v. those not critically ill/non-ICU cases and was associated with a poor prognosis. Subsequent studies confirmed these initial findings and also noted that other complications (such as ARDS and acute kidney injury) were more in those with cardiac injury than among those without cardiac injury.^[46]^ Furthermore, some studies noted cardiac involvement as a complication of COVID-19 infection even in cases with no symptoms and signs of pneumonia.^[47]^

Thereafter, a flurry of literature appeared questioning the role of the angiotensin-converting enzyme-2 receptor (ACE2) in the pathogenesis of these cardiac events in COVID-19 infection, with the recognition that ACE2 acted as a functional receptor for SARS-CoV-2, much like SARS-CoV before it, and even for influenza virus.^[48–53]^ This was fueled by the additional documentation that smoking and hypertension (the latter associated with the use of ACE inhibitors or ACE2 receptor blockers), both known risk factors for severe COVID-19 infection and mortality, were associated with an increased expression of ACE2. Furthermore, it was also documented that ACE2 expression was documented in cardiac muscle, suggesting a potential mechanism of cardiac injury.^[53]^ Concern was therefore raised about the use of angiotensin receptor blockers or ACE inhibitors, both from the point of view of the risk of COVID-19 infection, as well as the consideration as to what to do with these agents in patients taking them with an active COVID-19 infection. Fortunately, the various societies issued strong recommendations against the stopping of the use of these agents for treatment of patients with hypertension until more information was available, and this was supported in a special report in the *New England Journal of Medicine*. ^[54]^ This decision was subsequently justified by data from Wuhan, discounting the use of these agents as either a risk factor for severe disease or mortality from COVID-19.^[55]^ Lastly, with regard to drug therapy, it should be noted that several of the drugs that have been recommended as potential treatments for COVID-19 infection, including chloroquine/hydroxychloroquine, some antiviral agents and antibiotics such as the macrolides, both alone and even more so in combination, are potentially associated with risk of cardiac arrhythmias and need to be used with caution.^[56]^ The subject of potential effects of COVID-19 on the cardiovascular system and their management have been fully reviewed elsewhere.^[57,58]^ In addition, a European registry (CAPACITY-COVID) has been set up to accelerate knowledge of these cardiovascular events.^[59]^

While much of the previous discussion related mainly to cardiac events, it is also recognised that additional thrombotic events, both venous and arterial, do occur in patients with COVID-19 infection, especially in critically ill patients. Besides the cardiac events, these may include acute pulmonary embolism, deep venous thrombosis, ischaemic stroke and systemic arterial embolism and have been attributed to excessive inflammation, hypoxia, immobilisation, and diffuse intravascular coagulation and have been documented to occur in ~31% of cases.^[60,61]^ Pulmonary embolism appears to be the most common event^[60]^ and the presence of antiphospholipid antibodies associated with coagulopathy has been described as an additional possible mechanism in some cases.^[62]^ Recommendations have been published regarding prevention of these events in critically ill patients with COVID-19 infection, and while there is consensus that standard pharmacological prophylaxis should be given against thrombotic events, particularly in ICU cases, there has been some debate as to whether the standard doses recommended should be increased to high prophylactic doses or not, even if not clinically indicated, in the absence of randomised evidence.^[60,61]^

## Vaccination against CAP pathogens

We eagerly await an effective vaccine for the prevention of SARS CoV-2 infection; however, in the meanwhile there are potentially effective vaccines available for other common respiratory pathogens. These include the influenza and pneumococcal vaccines, which will assume even greater importance, as countries in the southern hemisphere, such as South Africa, move rapidly from summer into autumn and eventually into winter – a time when viruses increase in circulation and transmission and when pneumococcal infections reach their peak.^[63]^ While we do not know what will happen with the transmission of COVID-19 during the winter season, and the vaccines mentioned will not be protective against COVID-19 infection, they have the capacity to prevent CAP infections related to influenza and pneumococcus, and the cardiac complications associated with those infections, thus lessening the overall burden of respiratory infections in the population during this time period. The effectiveness of pneumococcal and influenza vaccination, alone and in combination, for the prevention of CAP and/or risk of cardiovascular events in patients with pneumococcal CAP, has been extensively reviewed elsewhere.^[9]^

## Conclusion

CAP remains an important cause of disease and death and recent research has highlighted the occurrence of cardiovascular events in patients with CAP that are associated with both short-term and long-term mortality. This includes infections with SARS-CoV-2, information about which is only emerging at the current time. A better understanding of the pathogenic mechanisms associated with these cardiovascular events may allow the development of novel treatment strategies and/or adjunctive therapies that may help improve patient outcomes.
